# Toward the Impact of English as a Foreign Language Teachers’ Grit and Self-Efficacy on Their Burnout

**DOI:** 10.3389/fpsyg.2022.876351

**Published:** 2022-06-01

**Authors:** Jinghan Zhou

**Affiliations:** College of International Education, Wenzhou University, Wenzhou, China

**Keywords:** teacher grit, teacher burnout, EFL teachers, mental state, teacher self-efficacy

## Abstract

Due to the adverse effects of teachers’ burnout on their professional performance, remarkable attention has been devoted to this mental state and its negative predictors. In this regard, multiple empirical research has been carried out to assess the effects of grit and self-efficacy as negative predictors of teacher burnout. Yet, no empirical or review study has delved into the impact of these variables at the same time. The current study attempts to fill this gap by delineating the impact of English as a Foreign Language (EFL) teachers’ grit and self-efficacy on their burnout. The significant role of grit and self-efficacy in curbing EFL teachers’ burnout was illustrated using theoretical and empirical evidence. The pedagogical implications are also offered.

## Introduction

Feelings of tension, stress, and anxiety are inherent challenges of demanding professions (Dewe et al., 2012), and teaching as a difficult vocation is not an exception by any means. These unpleasant feelings may lead to undesirable and negative consequences in the workplace. In classroom contexts, for instance, teachers who commonly experience prolonged, excessive stress and apprehension are prone to a negative state of mind called “burnout” (Fathi et al., 2021). Burnout as a psychological construct generally refers to “the state of physical and emotional depletion resulting from conditions of work” (Freudenberger, 1974, p. 161). Applied to the educational context, teacher burnout refers to a sense of “*emotional exhaustion*,” “*depersonalization*,” and “*reduced personal accomplishment*” that teachers may experience at a given point throughout their professional life (Maslach et al., 2001). As Maslach and Leiter (2016) noted, emotional exhaustion occurs when work overload and job-related stressors make teachers feel empty. Depersonalization also happens when teachers hold unfavorable and negative attitudes toward their vocation, their pupils, their colleagues, and the educational setting (Leiter and Maslach, 2016). Finally, reduced personal accomplishment pertains to a mental state that teachers perceive their teaching competence and abilities to be inadequate (Zhaleh et al., 2018). Put simply, this emotional state emerges when an individual teacher underestimates his/her own productivity (Seifalian and Derakhshan, 2018).

As put forward by Sabagh et al. (2018), teachers who feel emotionally and physically exhausted, hold negative viewpoints about their job, and underestimate their job-related capabilities are unable to effectively do the tasks they are responsible for. Put simply, an individual teacher who perceives himself/herself to be in a state of “burnout” cannot be a successful instructor anymore (Brasfield et al., 2019). This is due to the fact that the way teachers perform in classroom contexts is subjected to their mental and emotional state (Malmir and Mohammadi, 2008; Burić and Macuka, 2018; Greenier et al., 2021; Xie, 2021). Because of the adverse effects of burnout on teachers’ professional performance, factors preventing this mental state need to be discovered. To answer this necessity, numerous researchers have assessed the impact of emotional and psychological factors on teacher burnout (e.g., Vaezi and Fallah, 2011; Wang et al., 2015b; Lou and Chen, 2016; Tsang, 2018; Lu et al., 2019; Xu, 2019; Guan, 2020, to cite a few). Among different emotional and psychological factors, many scholars have focused on grit and self-efficacy, which are the focus of this study as well.

Grit generally refers to “one’s passion and perseverance for long term goals” (Duckworth et al., 2007, p. 1089). Extending this definition to the context of education, Robertson-Kraft and Duckworth (2014) described teacher grit as an individual teacher’s determination and persistence to lead his/her pupils to academic success. In the domain of second/foreign language education, teacher grit pertains to teachers’ perseverance and strong desire to guide their learners toward L2 success (MacIntyre and Khajavy, 2021). As Duckworth et al. (2009) mentioned, grit enables teachers to handle the challenges and difficulties of their profession, on the one hand, and to accomplish their profession-related responsibilities, on the other hand. That is, grittier teachers are more likely to succeed in their careers.

Another emotional factor that may negatively predict teacher burnout is self-efficacy, which generally pertains to “beliefs in one’s capabilities to organize and execute the courses of action required to produce given attainments” (Bandura, 1997, p. 4). In light of this conceptualization, Tschannen-Moran and Hoy (2001) defined teacher self-efficacy as “teachers’ judgment of their own capability to bring about desired outcomes of student learning, even among those students who may be difficult or unmotivated” (p. 783). Put simply, teachers’ sense of efficacy pertains to the degree to which a teacher thinks he/she has the potential to positively influence students’ learning outcomes (Fathi and Derakhshan, 2019). As Safari (2021) noted, a firm belief in professional capabilities empowers teachers to cope with the challenges of their vocation. Similarly, Shoji et al. (2016) submitted that self-efficacious teachers commonly show more perseverance against job-related stressors, pressures, and challenges.

Given the significant role of grit and self-efficacy in mitigating the causes of teacher burnout (Duckworth et al., 2009; Shoji et al., 2016), several researchers have empirically examined the effects of these two emotional and psychological factors on EFL teachers’ burnout (e.g., Federici and Skaalvik, 2012; Khezerlou, 2013; Wang et al., 2015a; Zhu et al., 2018; Fabelico and Afalla, 2020; Fathi and Saeedian, 2020; Salehizadeh et al., 2020, to cite a few). However, compared to teacher self-efficacy, less attention has been devoted to the impact of teacher grit. Furthermore, no empirical or review study has simultaneously focused on the impact of grit and self-efficacy on EFL teachers’ burnout. The current review study is an endeavor to fill this gap by focusing on the effects of EFL teachers’ grit and self-efficacy on their burnout.

### Teacher Grit

The concept of “grit” refers to “stamina one has for sticking with long-term, life goals despite difficulties, failures, or adversities” (Duckworth and Gross, 2014, p. 320). Put simply, it pertains to a person’s desire and tenacity in attaining long-term objectives (Duckworth, 2016). Accordingly, teacher grit refers to teachers’ perseverance and tenacity in carrying out their job-related duties (Dobbins, 2016). According to Argon and Kaya (2018), teacher grit relates to the degree to which teachers persevere through adversities, challenges, and difficulties of their profession. As Dale et al. (2018) mentioned, instructors with high levels of grit are more likely to pursue and thrive in their vocation.

### Teacher Self-Efficacy

The term “self-efficacy” pertains to a person’s positive evaluation of his or her own personal capabilities (Bandura, 1997). Put simply, self-efficacy relates to the degree to which an individual believes in his/her ability to successfully perform a particular action (Bong and Skaalvik, 2003). Taking this definition into account, Tschannen-Moran and Johnson (2011) described teacher self-efficacy as “the teacher’s belief in his or her capability to organize and execute courses of action required to successfully accomplish a particular teaching task in a given context” (p. 752). As a multidimensional construct, teacher self-efficacy include three main dimensions (Brouwers and Tomic, 2000): “*magnitude*,” “*generality*,” and “*strength*.” Magnitude, as the first dimension, pertains to the *degree of performance* an individual teacher feels he or she is capable of achieving. The second dimension, generality, refers to how far improvements in self-efficacy beliefs may be extended to other behaviors and contexts. Strength, as the last dimension, refers to the *firmness* of an individual teacher’s belief that he or she is able to perform a particular activity (Dellinger et al., 2008; Wang and Guan, 2020; Han and Wang, 2021).

### Teacher Burnout

Burnout has been broadly defined as “a psychological syndrome that develops in response to chronic emotional and interpersonal job stressors” (Maslach, 2015, p. 931). Relying on this definition, Skaalvik and Skaalvik (2017) defined teacher burnout as a negative state of mind that teachers experience due to job-related stressors, pressures, and challenges. According to Bakker et al. (2014), teachers who are suffering from this mental state are unwilling to devote adequate time and energy to their profession. Similarly, Ansari et al. (2022) stated that burnout negatively affects the way teachers perform in classroom contexts. That is, teachers who are in a state of burnout are unable to do their educational tasks effectively.

### The Impact of Grit and Self-Efficacy on EFL Teachers’ Burnout

To elucidate the role of self-efficacy beliefs in curbing burnout among EFL teachers, Kim and Burić (2020) stated that those EFL teachers who firmly believe their own abilities will not experience feeling of “reduced personal accomplishment,” which is one of the main aspects of burnout (Maslach and Leiter, 2016; Wang, 2017; Wang and Guan, 2020). In this regard, Safari (2021) also declared that EFL teachers with a high level of self-efficacy are capable of controlling stress, pressures, and challenges, all of which are the main causes of burnout (Jacobson, 2016). Besides, regarding the impact of grit on EFL teachers’ burnout, Robertson-Kraft and Duckworth (2014) maintained that EFL teachers’ perseverance and determination push them to pursue their job to attain their professional goals. To them, grittier teachers can effectively cope with the job-related stressors that make them feel empty (Leiter and Maslach, 2016).

## Empirical Studies

To date, a plethora of research has been done on the impact of EFL teachers’ self-efficacy on their burnout (e.g., Federici and Skaalvik, 2012; Khezerlou, 2013; Yazdi et al., 2014; Zhu et al., 2018; Fathi and Saeedian, 2020; Fathi et al., 2021; Han and Wang, 2021, to cite a few). For instance, Zhu et al. (2018) examined the effect of self-efficacy on Chinese teachers’ burnout. To this end, 1892 Chinese teachers were asked to fill two self-reported questionnaires. Assessing the correlations of the questionnaires, they found a negative association between Chinese teachers’ self-efficacy and burnout. The results of structural equation modeling also indicated that Chinese teachers’ self-efficacy can drastically influence their burnout. In another study, Fathi and Saeedian (2020) delved into the impact of self-efficacy beliefs on Iranian EFL teachers’ burnout. To do so, 213 EFL teachers were opted from different universities and institutes in Iran. They were invited to respond to two valid scales. Using structural equation modeling, the researchers found that teachers’ sense of efficacy can significantly predict their burnout in a negative way. Besides, some studies have also been performed on the effects of EFL teachers’ grit on their burnout (e.g., Fabelico and Afalla, 2020; Heruela, 2021). For instance, Fabelico and Afalla (2020) explored the influence of grit on Philippines teachers’ burnout. To this aim, 128 university teachers were asked to answer two reliable inventories. The results demonstrated that grit can negatively predict their Philippines teachers’ burnout.

## Conclusion

So far, the concepts of teacher grit, teacher self-efficacy, and teacher burnout were clearly described. Moreover, the dimensions and components of these variables were outlined. Furthermore, the role of grit and self-efficacy in reducing EFL teachers’ burnout was elucidated using theoretical and empirical evidence. Relying on the available evidence, one can logically conclude that both grit and self-efficacy can negatively predict EFL teachers’ burnout. Put simply, grittier and self-efficacious teachers are less likely to experience burnout during their professional life. This outcome seems insightful and instructive for all English language teachers in any EFL context. Given the importance of self-efficacy beliefs in curbing teacher burnout, EFL teachers should have faith in their professional abilities in order to energetically pursue their vocation. The finding of this review appears to be enlightening for teacher educators as well. As the outcomes of this review study revealed, teachers with strong desire and perseverance can handle the job-related stressors, which is the main cause of burnout. Accordingly, teacher educators need to teach their trainees how to persevere against the pressures, challenges, and problems of teaching profession.

## Author Contributions

The author confirms being the sole contributor of this work and has approved it for publication.

## Conflict of Interest

The author declares that the research was conducted in the absence of any commercial or financial relationships that could be construed as a potential conflict of interest.

## Publisher’s Note

All claims expressed in this article are solely those of the authors and do not necessarily represent those of their affiliated organizations, or those of the publisher, the editors and the reviewers. Any product that may be evaluated in this article, or claim that may be made by its manufacturer, is not guaranteed or endorsed by the publisher.

## References

[ref1] Ansari A. Pianta R. C. Whittaker J. V. Vitiello V. E. Ruzek E. A. (2022). Preschool teachers’ emotional exhaustion in relation to classroom instruction and teacher-child interactions. Early Educ. Dev. 33, 107–120. doi: 10.1080/10409289.2020.1848301

[ref2] Argon T. Kaya A. (2018). Examination of grit levels of teachers according to personal variables. J. Educ. Train. Stud. 6, 45–53. doi: 10.11114/jets.v6i3a.3157

[ref3] Bakker A. B. Demerouti E. Sanz-Vergel A. I. (2014). Burnout and work engagement: the JD–R approach. Annu. Rev. Organ. Psych. Organ. Behav. 1, 389–411. doi: 10.1146/annurev-orgpsych-031413-091235, PMID: 34899485

[ref4] Bandura A. (ed.) (1997). Self-Efficacy: The Exercise of Control. New York, NY: Freeman.

[ref5] Bong M. Skaalvik E. M. (2003). Academic self-concept and self-efficacy: how different are they really. Educ. Psychol. Rev. 15, 1–40. doi: 10.1023/A:1021302408382, PMID: 34708834

[ref6] Brasfield M. W. Lancaster C. Xu Y. J. (2019). Wellness as a mitigating factor for teacher burnout. J. Educ. 199, 166–178. doi: 10.1177/0022057419864525

[ref7] Brouwers A. Tomic W. (2000). A longitudinal study of teacher burnout and perceived self-efficacy in classroom management. Teach. Teach. Educ. 16, 239–253. doi: 10.1016/S0742-051X(99)00057-8

[ref8] Burić I. Macuka I. (2018). Self-efficacy, emotions and work engagement among teachers: a two wave cross-lagged analysis. J. Happiness Stud. 19, 1917–1933. doi: 10.1007/s10902-017-9903-9

[ref9] Dale G. Sampers D. Loo S. Green C. S. (2018). Individual differences in exploration and persistence: grit and beliefs about ability and reward. PLoS One 13:e0203131. doi: 10.1371/journal.pone.0203131, PMID: 30180200 PMC6122809

[ref10] Dellinger A. B. Bobbett J. J. Olivier D. F. Ellett C. D. (2008). Measuring teachers’ self-efficacy beliefs: development and use of the TEBS-self. Teach. Teach. Educ. 24, 751–766. doi: 10.1016/j.tate.2007.02.010

[ref11] Dewe P. J. O’Driscoll M. P. Cooper C. L. (2012). “Theories of psychological stress at work,” in Handbook of Occupational Health and Wellness. eds. Gatchel R. J. Schultz I. Z. (Switzerland: Springer), 23–38.

[ref12] Dobbins D. (2016). Teacher effectiveness: examining the relationship between teacher grit and teacher self-efficacy. Doctoral Dissertation. Stillwater, Oklahoma: Oklahoma State University.

[ref13] Duckworth A. (ed.) (2016). Grit: The Power of Passion and Perseverance. New York, NY: Scribner.

[ref14] Duckworth A. Gross J. J. (2014). Self-control and grit: related but separable determinants of success. Curr. Dir. Psychol. Sci. 23, 319–325. doi: 10.1177/0963721414541462, PMID: 26855479 PMC4737958

[ref15] Duckworth A. L. Peterson C. Matthews M. D. Kelly D. R. (2007). Grit: perseverance and passion for long-term goals. J. Pers. Soc. Psychol. 92, 1087–1101. doi: 10.1037/0022-3514.92.6.1087, PMID: 17547490

[ref16] Duckworth A. L. Quinn P. D. Seligman M. E. (2009). Positive predictors of teacher effectiveness. J. Posit. Psychol. 4, 540–547. doi: 10.1080/17439760903157232, PMID: 35126266

[ref17] Fabelico F. Afalla B. (2020). Perseverance and passion in the teaching profession: teachers’ grit, self-efficacy, burnout, and performance. J. Crit. Rev. 7, 108–119.

[ref18] Fathi J. Derakhshan A. (2019). Teacher self-efficacy and emotional regulation as predictors of teaching stress: an investigation of Iranian English language teachers. Teach. Engl. Lang. 13, 117–143. doi: 10.22132/TEL.2019.95883

[ref19] Fathi J. Greenier V. Derakhshan A. (2021). Self-efficacy, reflection, and burnout among Iranian EFL teachers: the mediating role of emotion regulation. Iran. J. Lang. Teach. Res. 9, 13–37. doi: 10.30466/IJLTR.2021.121043

[ref20] Fathi J. Saeedian A. (2020). A structural model of teacher self-efficacy, resilience, and burnout among Iranian EFL teachers. Iran. J. Engl. Acad. Purp. 9, 14–28.

[ref21] Federici R. A. Skaalvik E. M. (2012). Principal self-efficacy: relations with burnout, job satisfaction and motivation to quit. Soc. Psychol. Educ. 15, 295–320. doi: 10.1007/s11218-012-9183-5

[ref22] Freudenberger H. J. (1974). Staff burn-out. J. Soc. Issues 30, 159–165. doi: 10.1111/j.1540-4560.1974.tb00706.x, PMID: 35255580

[ref23] Greenier V. Derakhshan A. Fathi J. (2021). Emotion regulation and psychological well-being in teacher work engagement: a case of British and Iranian English language teachers. System 97:102446. doi: 10.1016/j.system.2020.102446

[ref24] Guan L. (2020). Research on job burnout of Chinese college English teachers in Sichuan province based on field rules theory. Theory Pract. Lang. Stud. 10, 313–317. doi: 10.17507/tpls.1003.07

[ref25] Han Y. Wang Y. (2021). Investigating the correlation among Chinese EFL teachers’ self-efficacy, reflection, and work engagement. Front. Psychol. 12:763234. doi: 10.3389/fpsyg.2021.763234, PMID: 34803845 PMC8603392

[ref26] Heruela K. S. G. P. (2021). Mediating effect of burnout on the relationship between grit and turnover intentions among the private tertiary school teachers in region XI: a convergent design. Int. J. Interdiscipl. Stud. 2, 73–90. doi: 10.51798/sijis.v2i4.178

[ref27] Jacobson D. A. (2016). Causes and effects of teacher burnout. Doctoral Dissertation. Minneapolis, Minnesota: Walden University.

[ref28] Khezerlou E. (2013). Teacher self-efficacy as a predictor of job burnout among Iranian and Turkish EFL teachers. Procedia Soc. Behav. Sci. 70, 1186–1194. doi: 10.1016/j.sbspro.2013.01.175

[ref29] Kim L. E. Burić I. (2020). Teacher self-efficacy and burnout: determining the directions of prediction through an autoregressive cross-lagged panel model. J. Educ. Psychol. 112, 1661–1676. doi: 10.1037/edu0000424

[ref30] Leiter M. P. Maslach C. (2016). Latent burnout profiles: a new approach to understanding the burnout experience. Burn. Res. 3, 89–100. doi: 10.1016/j.burn.2016.09.001

[ref31] Lou Y. Chen L. (2016). A study of the English teachers’ burnout in a local comprehensive university in China. Creat. Educ. 07, 646–654. doi: 10.4236/ce.2016.74067

[ref32] Lu M. H. Luo J. Chen W. Wang M. C. (2019). The influence of job satisfaction on the relationship between professional identity and burnout: a study of student teachers in Western China. Curr. Psychol. 41, 289–297. doi: 10.1007/s12144-019-00565-7

[ref33] MacIntyre P. Khajavy G. H. (2021). Grit in second language learning and teaching: introduction to the special issue. J. Psychol. Lang. Learn. 3, 1–6. doi: 10.52598/jpll/3/2/1

[ref34] Malmir A. Mohammadi P. (2008). Teachers’ reflective teaching and self-efficacy as predicators of their professional success: a case of Iranian EFL teachers. Res. Engl. Lang. Pedagogy 6, 117–138. doi: 10.30486/relp.2018.528818

[ref35] Maslach C. (2015). Psychology of burnout. Int. Encycl. Soc. Behav. Sci. 2, 929–932. doi: 10.1016/B978-0-08-097086-8.26009-1

[ref36] Maslach C. Leiter M. P. (2016). Understanding the burnout experience: recent research and its implications for psychiatry. World Psychiatry 15, 103–111. doi: 10.1002/wps.20311, PMID: 27265691 PMC4911781

[ref37] Maslach C. Schaufeli W. B. Leiter M. P. (2001). Job burnout. Annu. Rev. Psychol. 52, 397–422. doi: 10.1146/annurev.psych.52.1.397, PMID: 11148311

[ref38] Robertson-Kraft C. Duckworth A. L. (2014). True grit: trait-level perseverance and passion for long-term goals predicts effectiveness and retention among novice teachers. Teach. Coll. Rec. 116:25364065. doi: 10.1177/016146811411600306, PMID: 25364065 PMC4211426

[ref39] Sabagh Z. Hall N. C. Saroyan A. (2018). Antecedents, correlates and consequences of faculty burnout. Educ. Res. 60, 131–156. doi: 10.1080/00131881.2018.1461573

[ref40] Safari I. (2021). Relationship between Iranian EFL teachers’ self-efficacy and their burnout level in universities and schools. Int. J. Foreign Lang. Teach. Res. 9, 25–38.

[ref41] Salehizadeh S. Shabani M. Malmir A. (2020). Iranian journal of English for academic purposes professionalism: the perceptions of Iranian English teachers of competence and performance in language teaching. Iranian J. Engl. Acad. Purp. 9, 1–14.

[ref42] Seifalian M. Derakhshan A. (2018). The relationship between Iranian EFL teachers’ burnout and self-efficacy across English-related vs. non-English-related academic degrees. Int. J. Engl. Lang. Transl. Stud. 6, 99–110.

[ref43] Shoji K. Cieslak R. Smoktunowicz E. Rogala A. Benight C. C. Luszczynska A. (2016). Associations between job burnout and self-efficacy: a meta-analysis. Anxiety Stress Coping 29, 367–386. doi: 10.1080/10615806.2015.1058369, PMID: 26080024

[ref44] Skaalvik E. M. Skaalvik S. (2017). Dimensions of teacher burnout: relations with potential stressors at school. Soc. Psychol. Educ. 20, 775–790. doi: 10.1007/s11218-017-9391-0

[ref45] Tsang K. K. (2018). The structural causes of teacher burnout in Hong Kong. Chin. Educ. Soc. 51, 449–461. doi: 10.1080/10611932.2018.1570797

[ref46] Tschannen-Moran M. Hoy A. W. (2001). Teacher efficacy: capturing an elusive construct. Teach. Teach. Educ. 17, 783–805. doi: 10.1016/S0742-051X(01)00036-1

[ref47] Tschannen-Moran M. Johnson D. (2011). Exploring literacy teachers’ self-efficacy beliefs: potential sources at play. Teach. Teach. Educ. 27, 751–761. doi: 10.1016/j.tate.2010.12.005

[ref48] Vaezi S. Fallah N. (2011). The relationship between emotional intelligence and burnout among Iranian EFL teachers. J. Lang. Teach. Res. 2, 1122–1129. doi: 10.4304/jltr.2.5.1122-1129

[ref49] Wang Y. L. (2017). Construction elements and path of practical education model in universities. EURASIA J. Math. Sci. Technol. 13, 6775–6782. doi: 10.12973/ejmste/78525

[ref50] Wang Y. L. Guan H. F. (2020). Exploring demotivation factors of Chinese learners of English as a foreign language based on positive psychology. Rev. Argent. Clin. Psicol. 29, 851–861. doi: 10.24205/03276716.2020.116

[ref51] Wang H. Hall N. C. Rahimi S. (2015a). Self-efficacy and causal attributions in teachers: effects on burnout, job satisfaction, illness, and quitting intentions. Teach. Teach. Educ. 47, 120–130. doi: 10.1016/j.tate.2014.12.005

[ref52] Wang Y. Ramos A. Wu H. Liu L. Yang X. Wang J.. (2015b). Relationship between occupational stress and burnout among Chinese teachers: a cross-sectional survey in Liaoning, China. Int. Arch. Occup. Environ. Health 88, 589–597. doi: 10.1007/s00420-014-0987-9, PMID: 25256806

[ref53] Xie F. (2021). A study on Chinese EFL teachers’ work engagement: the predictability power of emotion regulation and teacher resilience. Front. Psychol. 12:735969. doi: 10.3389/fpsyg.2021.735969, PMID: 34512487 PMC8430242

[ref54] Xu L. (2019). Teacher–researcher role conflict and burnout among Chinese university teachers: a job demand-resources model perspective. Stud. High. Educ. 44, 903–919. doi: 10.1080/03075079.2017.1399261

[ref55] Yazdi M. T. Motallebzadeh K. Ashraf H. (2014). The role of teacher's self-efficacy as a predictor of Iranian EFL teacher's burnout. J. Lang. Teach. Res. 5, 1198–1204. doi: 10.4304/jltr.5.5.1198-1204

[ref56] Zhaleh K. Ghonsooly B. Pishghadam R. (2018). Effects of conceptions of intelligence and ambiguity tolerance on teacher burnout: a case of Iranian EFL teachers. J. Res. Appl. Linguist. 9, 118–140. doi: 10.22055/RALS.2018.13796

[ref57] Zhu M. Liu Q. Fu Y. Yang T. Zhang X. Shi J. (2018). The relationship between teacher self-concept, teacher efficacy and burnout. Teachers Teach. 24, 788–801. doi: 10.1080/13540602.2018.1483913, PMID: 34886493

